# Genetic profile of scrapie codons 146, 211 and 222 in the *PRNP* gene locus in three breeds of dairy goats

**DOI:** 10.1371/journal.pone.0198819

**Published:** 2018-06-07

**Authors:** Sotiria Vouraki, Athanasios I. Gelasakis, Panoraia Alexandri, Evridiki Boukouvala, Loukia V. Ekateriniadou, Georgios Banos, Georgios Arsenos

**Affiliations:** 1 Laboratory of Animal Husbandry, School of Veterinary Medicine, Faculty of Health Sciences, Aristotle University of Thessaloniki, Thessaloniki, Greece; 2 Veterinary Research Institute of Thessaloniki, Hellenic Agricultural Organization Demeter, Thessaloniki, Greece; 3 Scotland’s Rural College and The Roslin Institute, University of Edinburgh, Scotland, United Kingdom; University of Lincoln, UNITED KINGDOM

## Abstract

Polymorphisms at *PRNP* gene locus have been associated with resistance against classical scrapie in goats. Genetic selection on this gene within appropriate breeding programs may contribute to the control of the disease. The present study characterized the genetic profile of codons 146, 211 and 222 in three dairy goat breeds in Greece. A total of 766 dairy goats from seven farms were used. Animals belonged to two indigenous Greek, Eghoria (n = 264) and Skopelos (n = 287) and a foreign breed, Damascus (n = 215). Genomic DNA was extracted from blood samples from individual animals. Polymorphisms were detected in these codons using Real-Time PCR analysis and four different Custom TaqMan^®^ SNP Genotyping Assays. Genotypic, allelic and haplotypic frequencies were calculated based on individual animal genotypes. Chi-square tests were used to examine Hardy-Weinberg equilibrium state and compare genotypic distribution across breeds. Genetic distances among the three breeds, and between these and 30 breeds reared in other countries were estimated based on haplotypic frequencies using fixation index F_ST_ with Arlequin v3.1 software; a Neighbor-Joining tree was created using PHYLIP package v3.695. Level of statistical significance was set at P = 0.01. All scrapie resistance-associated alleles (146S, 146D, 211Q and 222K) were detected in the studied population. Significant frequency differences were observed between the indigenous Greek and Damascus breeds. Alleles 222K and 146S had the highest frequency in the two indigenous and the Damascus breed, respectively (*ca*. 6.0%). The studied breeds shared similar haplotypic frequencies with most South Italian and Turkish breeds but differed significantly from North-Western European, Far East and some USA goat breeds. Results suggest there is adequate variation in the *PRNP* gene locus to support breeding programs for enhanced scrapie resistance in goats reared in Greece. Genetic comparisons among goat breeds indicate that separate breeding programs should apply to the two indigenous and the imported Damascus breeds.

## Introduction

Scrapie is an infectious, neurodegenerative and fatal disease of sheep and goats. Along with bovine spongiform encephalopathy, Creutzfeldt-Jacob disease in humans and chronic wasting disease in cervids, scrapie belongs to the group of transmissible spongiform encephalopathies (TSEs), also known as prion diseases. Scrapie is the result of the accumulation of PrP^Sc^ in the central nervous system, which is an abnormal β-sheet rich isoform of the normal a-helix rich PrP^C^ protein [1].

In sheep, susceptibility to classical scrapie is modulated by the *PRNP* gene which encodes PrP^C^ protein [2]. Polymorphisms at codons 136 (A/V), 154 (Q/R) and 171 (Q/R/H) of this gene have been associated with susceptibility (ARQ and VRQ haplotypes) or resistance (ARR haplotypes) to classical scrapie [3,4,5,6]. Based on these findings a five-group risk classification system has been developed [7] and applied by different countries in selective breeding programs to control and eradicate the disease [8].

In goats, previous research has revealed more than 30 polymorphic codons in the *PRNP* gene. Amongst these, polymorphisms at codons 142 (I/M), 143 (H/R), 146 (N/S/D), 154 (H/R), 211 (R/Q) and 222 (Q/K) have been associated with resistance or susceptibility to clinical manifestation of the disease in field studies [9–16]. Experimental studies have shown that alleles 222K, 146S and 211Q confer the strongest degree of resistance after oral and/or intracerebral challenge and, therefore, are considered the most suitable candidates for selective breeding programs in goats [17–22]. This is also supported by the European Food Security Authority (EFSA) in their scientific opinion on the genetic resistance to TSEs in goats [23]. However, there are currently no formal regulations to underpin selective breeding programs in goats, equivalent to those in sheep. The notion is that success of such programs is subject to in-depth knowledge of the frequency and distribution of *PRNP* polymorphisms conferring resistance to classical scrapie [14,23]. Different estimates of allelic frequency have been published for breeds in China [24], Japan [25], USA [26], Italy [27–29], France [13], UK [30], Cyprus [31] and Turkey [32]. Given the observed differences between countries and breeds, EFSA concluded that any selective breeding programs should be developed and managed at national level, and in each case, the frequency of the resistance-associated alleles and haplotypes should be assessed for each breed of interest [23].

Dairy goats constitute a significant sector of livestock farming in Greece. The Greek goat population is approximately 3.8 million [33], which makes it the largest in Europe and one of the largest worldwide. This population is composed mainly of the Eghoria and Skopelos indigenous breeds, as well as the imported Damascus breed along with various types of crossbreds. Scrapie was first reported in Greece in 1986 and has since been considered a major health problem in both sheep flocks and goat herds. Studies on the frequencies of classical scrapie resistance-associated alleles in goats of Greece are limited [15,34,35]. These studies either focused on a small number of goats from scrapie-affected herds [34] or animals of unspecified breeds [15,35]. A systematic large-scale population study of the *PRNP* gene polymorphisms in dairy goat breeds reared in Greece is missing.

The objective of the present study was to assess the genetic profile of codons 146, 211 and 222 in the *PRNP* gene locus in the three key dairy goat breeds in Greece, namely Eghoria, Skopelos and Damascus in order to determine the feasibility of breeding programs aiming at enhancing resistance to classical scrapie.

## Materials and methods

### Ethics statement

This study followed the European Directive 86/609/EEC and its national implementation in Greece Presidential Decree No 160/1991 (Governmental Gazette No A' 64). Blood sampling of dairy goats was performed in commercial farms within the EU SOLID project (FP7-KBBE-266367). This research was approved by the Research Committee of The Aristotle University of Thessaloniki (26362/03.05.2011).

### Animals

A total of 766 dairy goats of two indigenous Greek breeds (Eghoria and Skopelos, n = 264 and n = 287 goats, respectively) and one foreign breed (Damascus, n = 215 goats) were used. Animals of 1–4 years of age were randomly selected from seven farms (two with Eghoria, two with Skopelos and three with Damascus goats). Goat herds were selected as representatives of the prevailing farming systems in the country based on the *a posteriori* typology scheme described by Gelasakis *et al*. [36]. These were low-input pastoral farming systems characterized by grazing throughout the year, random mating and minor differences in management practices between herds [36]. Based on the latter, the selected number of seven herds was considered sufficient for the study. Relations of the selected animals were unknown since in all cases, random mating was performed. However, considering that the number of bucks and their replacement rate were high, over-representation of lineages is unlikely.

### Genotyping

Blood samples were collected from the jugular vein in EDTA vacutainers and genomic DNA was extracted using GeneJET Whole Blood Genomic DNA Purification Mini Kit (Thermo Scientific, Waltham, Massachusetts, USA) according to manufacturer’s instructions. Polymorphisms at codons 146 (N/S/D), 211 (R/Q) and 222 (Q/K) were detected using a novel genotyping approach. Contrary to previous studies, which used PCR amplification of the open reading frame of caprine *PRNP* gene and sequencing of the PCR products, we focused on the most important codons (146, 211 and 222) and developed four separate Real-Time PCR reactions with four different Custom TaqMan^®^ Single Nucleotide Polymorphism (SNP) Genotyping Assays (Applied Biosystems, Foster City, California, USA). Real-Time PCR is fast, sensitive and suitable for SNP detection. This method has been also used in sheep for the detection of polymorphisms at codons 136, 154 and 171 in the *PRNP* gene [37]. Each SNP Genotyping Assay consisted of a mix of sequence-specific forward and reverse primers to amplify the polymorphic sequence of interest and two TaqMan^®^ MGB (minor groove binder) probes, FAM (6-carboxyfluorescein) and VIC (2′-chloro-7′phenyl-1,4-dichloro-6-carboxy-fluorescein) dye-labeled to detect the amplified product (Table 1). Primers and probes were designed by Applied Biosystems using designated software and then individually tested by mass spectroscopy to verify the accuracy of the resulting synthesized oligonucleotide. Validation of the genotyping method was performed using sequenced goat genomic DNA samples with all possible genotypes at codons 146, 211 and 222.

**Table 1:** Primer and probe sequences used in four Custom TaqMan® SNP Genotyping Assays for the detection of polymorphisms 146 (N/S), 146 (N/D), 211 (R/Q) and 222 (Q/K).

SNP	Primer	
	Forward	Reverse
146(N/S)	GCCATGAGCAGGCCTCTTATA	GGGTAACGGTACATGTTTTCACGAT
146(N/D)	GCCATGAGCAGGCCTCTT	GGGTAACGGTACATGTTTTCACGAT
211(R/Q)	GAACTTCACCGAAACTGACATCAAG	ACTGGGTGATGCACATTTGCT
222(Q/K)	TGGTGGAGCAAATGTGCATCA	GGGAAGAAAAGAGGATCACACTTG
	Probe^†^	
	FAM	VIC
146(N/S)	TTTTGGCAGTGACTATG	CATTTTGGCAATGACTATG
146(N/D)	CATTTTGGCAATGACT	ATACATTTTGGCGATGACT
211(R/Q)	AATGGAGCAAGTGGTG	ATAATGGAGCGAGTGGTG
222(Q/K)	CTGGGATTCTCTCTTGTACTG	TGGGATTCTCTCTGGTACTG

^†^In all cases except for 146(N/D), FAM dye-labeled probes were used for the detection of the mutated sequences and VIC dye-labeled probes for the detection of the wildtype sequences.

PCR reactions were performed in 12.5 μl mixtures containing 6.25 μl of KAPA PROBE FAST qPCR Master Mix (2X) Universal (Kapa Biosystems, Wilmington, Massachusetts, USA), 0.3125 μl of each SNP Genotyping Assay Mix (40X) and 1 μl (*ca*. 50 ng) of genomic DNA. Thermal cycling included: a) an initial denaturation step at 95°C for 3 min and b) 45 cycles of denaturation at 95°C for 3 s and primer annealing/extension at 62°C for 30 s. All Real-Time PCR analyses (n = 3,064) were performed using Applied Biosystems StepOnePlus^TM^ Real-Time PCR System and genotypes were determined through amplification plots with QPCR Applied Biosystems Step One Software v2.3 (dx.doi.org/10.17504/protocols.io.qeidtce).

### Genetic analyses

Genotypic, allelic and haplotypic frequencies regarding codons 146, 211 and 222 were calculated within and across breed, based on counting of the respective genotypes of individual animals. Hardy-Weinberg equilibrium state was examined at each codon and breed using a chi-square test:

$$\chi^{2}=\sum \left[{\left(O–E\right)}^{2}/E\right]$$

Where

O is the observed number of each genotype;

E is the expected number of each genotype assuming Hardy-Weinberg equilibrium;

Summation is over all possible genotypes.

Pairwise comparison of genotypic distribution between breeds at each codon was carried out using a chi-square test. In each case, number of observed genotypes in one breed was compared to expected number based on the allelic frequency in the other breed. Moreover, genetic distances among the three studied breeds were estimated on the basis of haplotypic frequencies using fixation index F_ST_ and Arlequin v3.1 software [38]. To add substance to these comparisons, genetic distances were also calculated between the three studied breeds and 30 other goat breeds from different countries, for which haplotypic frequencies were available in the existing literature (S1 Table). In each case, the significance of difference from the null value was tested with 1,000 permutations. The matrix of genetic distances between breed pairs was used to create a Neighbor-Joining tree deploying the PHYLIP package v3.695 [39].

In all the above analyses, level of statistical significance was set at P = 0.01.

## Results

### Frequencies

Genotypic, allelic and haplotypic frequencies in the *PRNP* gene locus are presented in Tables 2 and 3 and S2 Table, respectively. Across all breeds, four out of six possible genotypes at codon 146 (NN, NS, ND and SS), and two out of three at each of codons 211 (RR and RQ) and 222 (QQ and QK) were detected. Genotypes carrying resistance-associated alleles, namely 222QK, 146NS, 211RQ, 146ND and 146SS were found at a frequency of 8.74%, 3.39%, 2.87%, 0.13% and 0.13%, respectively (Table 2). All resistance-associated alleles were observed in the three breeds collectively; the most frequent was 222K (4.37%), followed by 146S (1.83%), 211Q (1.44%) and 146D (0.06%, Table 3). Within breed, allele 222K had the highest frequency in the two indigenous Greek breeds (5.87% and 5.92% in Eghoria and Skopelos, respectively), whereas 146S was the most frequent in Damascus (6.05%, Table 3). Frequencies of the resistance-associated polymorphisms dictated the estimation of haplotypic frequencies in the *PRNP* gene locus; haplotypes carrying multiple resistance-associated polymorphisms were not detected (S2 Table).

**Table 2:** Genotypic frequencies (%) and corresponding number of animals (in parenthesis) at codons 146, 211 and 222 of the *PRNP* gene locus.

		Breed			
Codon	Genotype	Eghoria^a^	Skopelos^a^	Damascus^b^	Total
146	NN	99.62 (263)	99.65 (286)	87.91 (189)	96.35 (738)
	NS	0.00 (0)	0.35 (1)	11.63 (25)	3.39 (26)
	SS	0.00 (0)	0.00 (0)	0.46 (1)	0.13 (1)
	ND	0.38 (1)	0.00 (0)	0.00 (0)	0.13 (1)
	DD	0.00 (0)	0.00 (0)	0.00 (0)	0.00 (0)
	DS	0.00 (0)	0.00 (0)	0.00 (0)	0.00 (0)
211	RR	96.97 (256)	100.00 (287)	93.49 (201)	97.13 (744)
	RQ	3.03 (8)	0.00 (0)	6.51 (14)	2.87 (22)
	QQ	0.00 (0)	0.00 (0)	0.00 (0)	0.00 (0)
222	QQ	88.26 (233)	88.15 (253)	99.07 (213)	91.26 (699)
	QK	11.74 (31)	11.85 (34)	0.93 (2)	8.74 (67)
	KK	0.00 (0)	0.00 (0)	0.00 (0)	0.00 (0)

^a,b^ Comparison of genotypic distributions between breeds; different superscripts denote significant differences (P<0.01); distributions were compared within codon and outcome was the same in all three codons.

**Table 3:** Allelic frequencies (%) at codons 146, 211 and 222 of the *PRNP* gene locus.

		Breed			
Codon	Allele	Eghoria	Skopelos	Damascus	Total
146	N	99.81	99.83	93.95	98.11
	S*	0.00	0.17	6.05	1.83
	D*	0.19	0.00	0.00	0.06
211	R	98.48	100	96.74	98.56
	Q*	1.52	0.00	3.26	1.44
222	Q	94.13	94.08	99.53	95.63
	K*	5.87	5.92	0.47	4.37

*Indicates alleles which are considered to confer resistance to scrapie.

All three codons were found to be in Hardy-Weinberg equilibrium (P>0.01) in all breeds, suggesting absence of direct or indirect genetic selection pressure.

### Genetic comparison among the three goat breeds in Greece

Genotypic distribution in Damascus breed differed significantly (P<0.01) from that in Eghoria and Skopelos breeds for each of the three codons (Table 2). No significant differences (P>0.01) were observed between Eghoria and Skopelos breeds in any codon.

Genetic distance analysis (Table 4) showed that Eghoria and Skopelos breeds shared similar haplotypic frequencies implying genetic relatedness (F_ST_ = 0.000, P>0.01), whereas they differed significantly from Damascus breed (F_ST_ = 0.020, P<0.001 and F_ST_ = 0.028, P<0.001, respectively), thereby corroborating the genotypic distribution results.

**Table 4:** Genetic (F_ST_) distances (above diagonal) and corresponding P-values (below diagonal) among Eghoria, Skopelos and Damascus breeds.

Breeds	Eghoria	Skopelos	Damascus
Eghoria		0.000	0.020
Skopelos	0.31543		0.028
Damascus	<0.00001*	<0.00001*	

*Indicates significant P-values (P<0.01); five decimal points is the maximum number provided by the Arlequin software.

### Genetic comparison between the three goat breeds in Greece and breeds in other countries

Estimated genetic distances between goat breeds reared in Greece and in other countries are presented in Table 5. Small F_ST_ estimates and high (non-significant) P-values denote high levels of genetic relatedness between respective breeds. The three studied breeds shared similar haplotypic frequencies (P>0.01) with most breeds reared in neighboring Mediterranean regions (Southern Italy, Turkey and Cyprus). Notable exceptions were the observed distances (P<0.01) from four Southern Italian breeds (Garganica, Girgentana, Derivata di Siria and Pantellaria), and the Damascus breed of Cyprus and Turkey. Significant genetic distances (P<0.01) were also observed from goat breeds reared in North-Western Europe (Northern Italy, France and UK) and Far East (Japan and China). Regarding breeds from the USA, Greek goats were genetically distant (P<0.01) from LaMancha and Nubian, while a significant difference was also observed between Skopelos and Alpine and Saanen breeds.

**Table 5:** Genetic (F_ST_) distances and corresponding P-values between goat breeds reared in Greece and other countries.

	Breeds in Greece					
	Eghoria		Skopelos		Damascus	
Other breeds (Region/Country^†^)	F_ST_	P-value	F_ST_	P-value	F_ST_	P-value
Garganica (Southern IT)	0.058	<0.00001*	0.076	<0.00001*	0.082	<0.00001*
Maltese (Southern IT)	0.009	0.19824	0.018	0.09961	0.037	0.01562
Ionica (Southern IT)	0.000	0.90723	0.000	0.78711	0.018	0.07812
Red Mediterranean (Southern IT)	0.000	0.77441	0.000	0.99902	0.016	0.06445
Girgentana (Southern IT)	0.064	<0.00001*	0.079	<0.00001*	0.087	<0.00001*
Derivata Di Siria (Southern IT)	0.034	<0.00001*	0.044	<0.00001*	0.060	<0.00001*
Pantellaria (Southern IT)	0.169	<0.00001*	0.216	<0.00001*	0.119	<0.00001*
Camosciata Delle Alpi (Northern IT)	0.059	<0.00001*	0.087	<0.00001*	0.040	<0.00001*
Saanen (Northern IT)	0.030	0.00098*	0.051	<0.00001*	0.021	0.00879*
Roccaverano (Northern IT)	0.063	<0.00001*	0.093	<0.00001*	0.046	<0.00001*
Valdostana (Northern IT)	0.027	<0.00001*	0.047	<0.00001*	0.017	0.00390*
Damascus (CY)	0.037	<0.00001*	0.046	<0.00001*	0.011	0.00293*
Damascus (TR)	0.263	<0.00001*	0.307	<0.00001*	0.159	<0.00001*
Akkeci (TR)	0.043	0.04980	0.074	0.01465	0.018	0.12793
Saanen (TR)	0.002	0.24805	0.013	0.06250	0.007	0.12109
Kilis (TR)	0.024	0.01171	0.031	0.01170	0.023	0.01855
Alpine (FR)	0.018	<0.00001*	0.032	<0.00001*	0.025	<0.00001*
Saanen (FR)	0.099	<0.00001*	0.127	<0.00001*	0.078	<0.00001*
Dairy breeds (UK)	0.027	<0.00001*	0.025	<0.00001*	0.042	<0.00001*
Boer (CN)	0.612	<0.00001*	0.656	<0.00001*	0.518	<0.00001*
Saanen (JP)	0.053	<0.00001*	0.077	<0.00001*	0.027	<0.00001*
Alpine (USA)	0.030	0.01465	0.052	0.00488*	0.010	0.12109
Oberhasli (USA)	0.012	0.09570	0.024	0.05371	0.007	0.17480
Toggenburg (USA)	0.000	0.86426	0.000	0.35742	0.010	0.08105
LaMancha (USA)	0.176	<0.00001*	0.212	<0.00001*	0.093	<0.00001*
Nubian (USA)	0.317	<0.00001*	0.360	<0.00001*	0.214	<0.00001*
Saanen (USA)	0.013	0.01367	0.019	0.00391*	0.007	0.07227

^†^IT = Italy, CY = Cyprus, TR = Turkey, FR = France, UK = United Kingdom, CN = China, JP = Japan, USA = United States of America

*Indicates significant P-values (P<0.01); five decimal points is the maximum number provided by the Arlequin software.

**Fig 1:**
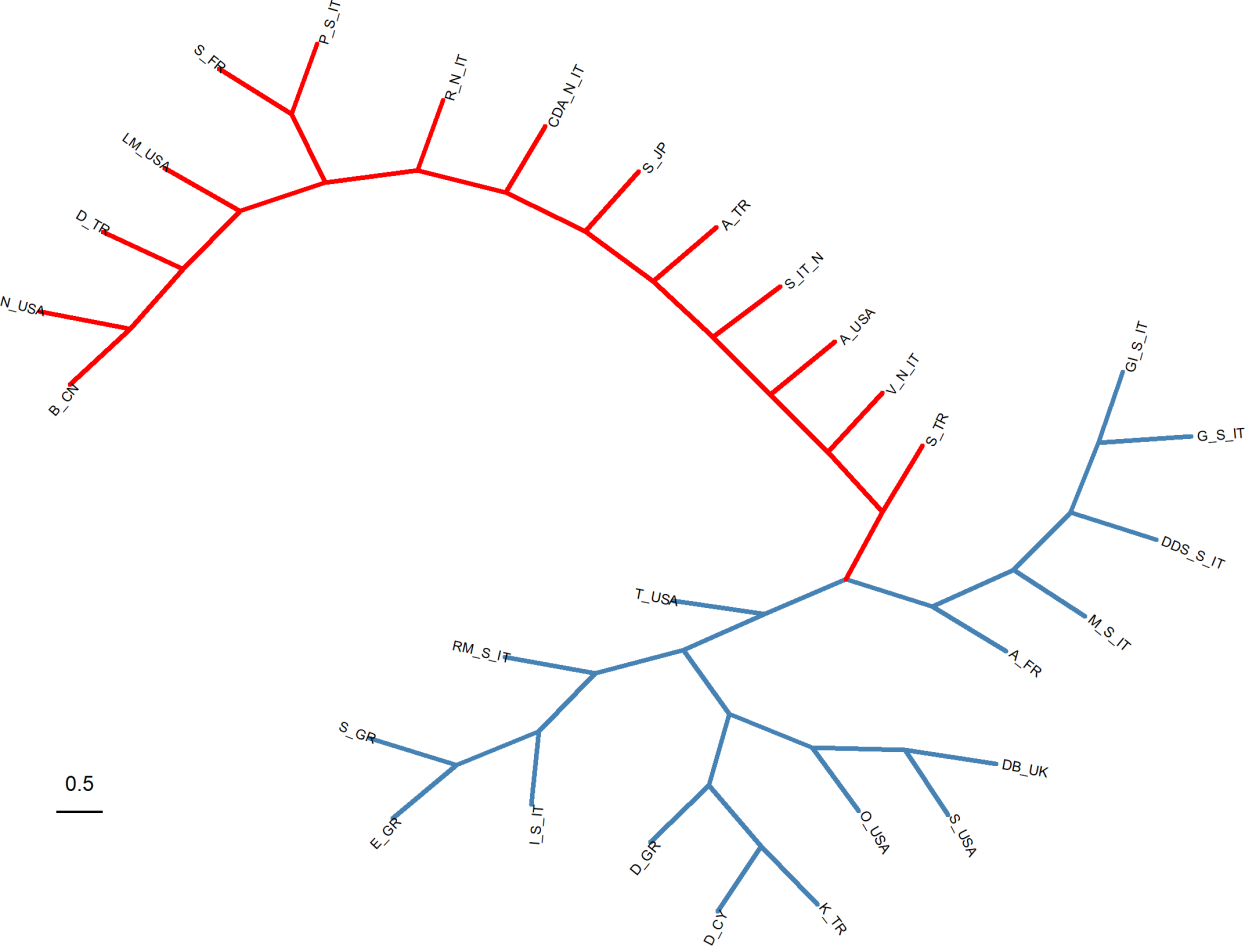
Unrooted Neighbor-Joining tree based on F_ST_ genetic distances. Colors indicate two major geographical clusters: Blue cluster includes: E_GR, Eghoria (Greece); S_GR, Skopelos (Greece); I_S_IT, Ionica (Southern Italy); RM_S_IT, Red Mediterranean (Southern Italy); D_GR, Damascus (Greece); D_CY, Damascus (Cyprus); K_TR, Kilis (Turkey); O_USA, Oberhasli (United States of America); S_USA, Saanen (United States of America); DB_UK, Dairy breeds (United Kingdom); T_USA, Toggenburg (United States of America); A_FR, Alpine (France); M_S_IT, Maltese (Southern Italy); DDS_S_IT, Derivata Di Siria (Southern Italy); G_S_IT, Garganica (Southern Italy); GI_S_IT, Girgentana (Southern Italy). Red cluster includes: S_TR, Saanen (Turkey); V_N_IT, Valdostana (Northern Italy); A_USA, Alpine (United States of America); S_IT_N, Saanen (Northern Italy); A_TR, Akkeci (Turkey); S_JP, Saanen (Japan); CDA_N_IT, Camosciata Delle Alpi (Northern Italy); R_N_IT, Roccaverano (Northern Italy); P_S_IT, Pantellaria (Southern Italy); S_FR, Saanen (France); LM_USA, La Mancha (United States of America), D_TR, Damascus (Turkey); N_USA, Nubian (United States of America); B_CN, Boer (China).

Results in Table 5 are broadly consistent with the Neighbor-Joining tree presented in Fig 1. The latter revealed two major geographical clusters; one including the three studied breeds and most breeds from other Mediterranean countries, and another including most of the North-Western European and the Far East breeds. Goat breeds from the USA were distributed across both clusters.

## Discussion

In the present study, the genetic profile of three codons (146, 211 and 222) in the *PRNP* gene locus was characterized for the first time in the three most common dairy goat breeds in Greece (Eghoria, Skopelos and Damascus). Our results demonstrated the presence of polymorphism in this locus which renders it amenable to change via genetic selection. Moreover, differences in *PRNP* haplotypic frequencies were revealed among the studied goat breeds but also between them and breeds reared in other countries, suggesting the importance of breeding programs being custom-designed according to the genetic profile of specific populations. In this regard, our results indicate that separate breeding programs should apply to the two indigenous and the imported Damascus breeds aiming at enhancing scrapie resistance.

Although resistance-associated allele frequencies have been previously reported in Greek goats, this is the first large-scale population study involving animals from specific breeds, without clinical signs of scrapie and from farms representative of the major farming systems as described in relevant typologies [36]. Regarding the Eghoria and Skopelos breeds, a previous study reported allele frequencies for 222K (*ca*. 24.0% in Skopelos) and 211Q (*ca*. 7.0% in both breeds) using goats from one highly infected herd [34]. However, these results are not directly comparable with ours as they were based on a different study design and a smaller sample size. Kanata *et al*. [35] reported frequencies in Greek goats higher than our study (3.0%, 0.5%, 6.0% and 5.6% for 146S, 146D, 211Q and 222K alleles, respectively) but for fewer animals (436 individuals) of unspecified breeds. Contrary to the latter study, we used purebred goats of the three most common dairy goat breeds reared in Greece.

The present study confirms that the dairy goat population in Greece, collectively represented here by the three studied breeds, is one of the very few worldwide where all scrapie resistance-associated alleles in the three codons (146, 211 and 222) have been detected. Previous research in Turkey has also reported presence of all these alleles in the goat population; however, much lower frequencies have been reported for alleles 222K and 211Q [32]. Polymorphic variation in the *PRNP* gene locus reported in our study enables the design and implementation of breeding programs towards enhanced scrapie resistance in goats reared in Greece.

The genetic profiles of scrapie codons 146, 211 and 222, revealed certain differences between the studied breeds. The most common resistance-associated allele was 222K in Eghoria and Skopelos (indigenous Greek breeds), whereas 146S in Damascus (*ca*. 6.0% in all cases). Such observations were further supported by comparison of genotypic distributions and genetic distance analyses, which showed significant differences between the two indigenous Greek breeds and Damascus. The latter is an imported breed of Middle East origin. Previous studies have suggested that geographical origin may explain a large part of genetic variability among goat breeds [39]. In a microsatellite analysis of European and Middle Eastern goats, Greek breeds were placed in the Central-Mediterranean cluster within which genetic differences among breeds were found to be relatively low [40]. In the same study, breeds of Middle Eastern origin were placed in a separate, East-Mediterranean genetic cluster [40].

To further investigate differences in the *PRNP* gene locus among different goat populations, we estimated the genetic distance between the studied breeds and 30 other breeds reared in various countries. To the best of our knowledge this is the first time that a large-scale comparison of the *PRNP* gene profile is attempted using collectively all the available data regarding polymorphism frequencies at codons 146, 211 and 222 in different dairy goat populations worldwide. In this regard, we derived novel and interesting results about the level of relatedness of these populations. Of course a certain level of caution needs to be exercised when interpreting these results as the relatively small number of polymorphisms and occasionally limited representation in the studied breeds might have influenced the accuracy of the genetic distance estimates. Furthermore, some of the previous studies on breeds reared in Italy, Turkey and USA had been based on just a few animals, which might have affected the representativeness of the calculated haplotypic frequencies.

According to previous research, allelic frequencies vary across countries and breeds [13, 24–32]. Results from our comparisons suggest that geographical origin of the breeds combined with population structure, breeding schemes and scrapie incidence seem to be the key determinants of the *PRNP* gene profile. Thus, the studied breeds shared similar haplotypic frequencies with many goat breeds reared in neighboring Mediterranean countries, such as certain breeds in Southern Italy (Maltese, Ionica and Red Mediterranean) [27] and Turkey (Saanen, Akkeci and Kilis) [32]. However, significant genetic distances were also detected in respect to some other breeds reared in neighboring countries. For example, much higher allelic frequencies of 222K were observed in Girgentana (18.7%) and Derivata Di Siria (15.0%), but the allele was absent in Pantellaria [28,29]. Girgentana is an endangered breed of Sicily with a small population size in which significant inbreeding and genetic drift have been recorded [28] potentially leading to this high allelic frequency. Derivata Di Siria goats are reared in a high-scrapie endemic area of Sicily, whereas Pantellaria goats are found in an area where scrapie has never been detected [29], quite possibly explaining the corresponding allelic frequencies in each case. Moreover, Pantellaria goats had allele 211Q in high frequency (23.0%) [29]. This could be interpreted as a consequence of the extensive crossbreeding with Saanen and Alpine goats of Northern Italy [29] in which allele 211Q was also found to be very common [27].

Furthermore, significant genetic distances were also detected between Greek Damascus and goats of the same breed in Turkey and Cyprus. In both Greek and Cypriot Damascus goats, 146S was the most common allele (*ca*. 6.0% to 7.0%) [31]. However, allele 146D, which was not detected in the present study, was also reported in Cyprus. In Damascus goats of Turkey, allele 146D was not detected, but a much higher frequency of 146S (*ca*. 28.0%) was reported [32]. Moreover, alleles 211Q and 222K were detected for the first time in Damascus goats of Greece. These results suggest a diverse evolution pattern of the Damascus breed in the three neighboring countries, where different breeding schemes, possibly involving crossbreeding with indigenous breeds, have taken place. Within-breed differences in haplotypic frequencies were also detected between Saanen goats reared in different countries (France, Italy, Turkey and USA) further enhancing the notion of diverse selective breeding in different localities.

All goat breeds in the present study were significantly distant from North-Western European and Far East breeds in which the most common alleles were 211Q (Northern Italian, French Alpine, French Saanen and Japanese Saanen breeds) or 146S (Chinese Boer), whereas 222K was either not detected or found in very low frequencies [13,24,25,27]. These findings could be explained by the different geographical origin of the breeds and are in agreement with the phylogeographical structure described by Canon *et al*. [40], which placed Northern Italian and French breeds in a different genetic cluster than the Greek ones. Our findings are also in agreement with previous studies of Italian goat breeds [27], where Northern Italian breeds were clustered separately from Southern Italian; the latter shared similar allelic frequencies with the Greek goats.

The three breeds of the present study had similar haplotypic frequencies with some breeds from USA. Such results might have been unexpected given that the latter were imported into USA from North-Western Europe. However, the sample size in these breeds on which haplotypic frequencies had been based was relatively small [26], which might have compromised the significance of the comparisons. Furthermore, no genetic scrapie studies of native USA goat breeds were found in the literature. Based on the phylogenetic analysis of goat mitochondrial sequences reported by Amills *et al*. [41], significant differences between native American and European populations could be expected.

All genetic comparisons among goat breeds discussed above support the scientific opinion of EFSA, which suggests that selective breeding programs towards scrapie resistance should be developed and managed independently within each country and according to established frequencies of resistance-associated alleles for each breed [23]. Therefore, our results suggest that separate breeding programs would be advisable for the Eghoria and Skopelos breeds on the one hand, and Damascus on the other. In all cases, however, caution should be exercised as uni-dimensional selection towards enhancing scrapie resistance could potentially lead to reduction of genetic variability and increased inbreeding, especially given the relatively low population frequencies of resistance-associated alleles [42]. An evidence-based approach to determine the best strategy is necessary. In a previous study in sheep, the impact of various selection strategies on inbreeding was investigated by simulating different frequencies of the resistant ARR haplotype and no negative effect was found when haplotypic frequency was at least 5.0% [43]. Moreover, in Chios Greek sheep, ARR haplotype was found at a frequency of 6.9% [44], which enabled the design of a selective breeding program towards enhancing scrapie resistance. This program is currently being implemented. Furthermore, Cyprus was able to start a breeding program for goat scrapie resistance focused on alleles 146S and 146D with frequencies of *ca*. 6.0% in the goat population [23]. Based on the above, a similar program for goat breeds reared in Greece is feasible. Such a program should aim at increasing the frequency of 222K allele in Eghoria and Skopelos goats, and the frequencies of 146S and, potentially, 211Q alleles in Damascus goats.

However, before such breeding programs are implemented, future research should address potentially adverse effects of genetic selection for enhanced scrapie resistance on other important animal traits. There might be a direct impact of *PRNP* gene in the phenotypic expression of such traits and/or genetic linkage between the *PRNP* gene and genes influencing animal performance [42]. Although relevant studies have been published for sheep [44–49], studies on goats are missing.

Finally, a critical step towards establishing effective breeding programs is to fully understand the allelic interactions in all codons of interest. A partial dominant effect of allele 222K over the wild-type was experimentally detected by intra-cerebrally inoculating mice with various scrapie isolates; all KK animals were resistant and QK were more resistant than QQ animals showing reduced incidence rates and/or longer incubation periods [19]. To the best of our knowledge, no relevant studies have been published for alleles in other codons. Therefore, experimental goat studies including both heterozygous and homozygous carriers of resistance-associated alleles are warranted.

## Conclusions

The results of the present study indicate that there is adequate variation in the *PRNP* gene locus to support breeding programs for enhanced scrapie resistance in goats reared in Greece. According to the genetic profile in the three studied codons, separate breeding programs should apply to the indigenous breeds and the imported Damascus breed. Future breeding programs in other countries and breeds should first study the genetic profile of the goat population in question. However, before any measures are taken it is necessary to: i) determine possible adverse effects of selection towards scrapie resistance on other important animal traits and ii) fully understand the allelic interactions in the involved codons.

## Supporting information

S1 Dataset Genotypes at codons 146, 211 and 222 of the *PRNP* gene locus of individual Eghoria, Skopelos and Damascus goats obtained in the study. (XLSX)

S1 Table Goat breeds with corresponding number of animals and haplotypic frequencies (%) at the *PRNP* gene locus (codon order 146, 211, 222) by country and literature reference. (DOCX)

S2 Table Haplotypic frequencies (%) at the *PRNP* gene locus (codon order 146, 211, 222). (DOCX)
