# Supplementary material for: Genetic profile of scrapie codons 146, 211 and 222 in the PRNP gene locus in three breeds of dairy goats
Source: PLoS One. 2018 Jun 7;13(6):e0198819. doi: 10.1371/journal.pone.0198819 (PMC5991713; doi:10.1371/journal.pone.0198819)
Supplement: S1 Table — (DOCX) [file pone.0198819.s002.docx]

**S1 Table. Goat breeds with corresponding number of animals and haplotypic frequencies (%) at the *PRNP* gene locus (codon order 146, 211, 222) by country and literature reference.**

| **Breed** | **Country** | **Animals (no)** | **Haplotype** | **Frequency** | **Reference** |
| --- | --- | --- | --- | --- | --- |
| Garganica | Italy | 58 | NRQ | 82.80 | [27] |
|  |  |  | NRK | 17.20 |  |
| Maltese | Italy | 25 | NRQ | 88.00 | [27] |
|  |  |  | NRK | 12.00 |  |
| Ionica | Italy | 27 | NRQ | 92.70 | [27] |
|  |  |  | NRK | 7.30 |  |
| Red Mediterranean | Italy | 28 | NRQ | 94.60 | [27] |
|  |  |  | NRK | 5.40 |  |
| Camosciata Delle Alpi | Italy | 84 | NRQ | 83.90 | [27] |
|  |  |  | NQQ | 13.70 |  |
|  |  |  | NRK | 2.40 |  |
| Saanen | Italy | 69 | NRQ | 86.80 | [27] |
|  |  |  | NQQ | 10.20 |  |
|  |  |  | NRK | 3.00 |  |
| Roccaverano | Italy | 70 | NRQ | 82.10 | [27] |
|  |  |  | NQQ | 13.60 |  |
|  |  |  | NRK | 4.30 |  |
| Valdostana | Italy | 77 | NRQ | 89.10 | [27] |
|  |  |  | NQQ | 9.60 |  |
|  |  |  | NRK | 1.30 |  |
| Girgentana | Italy | 158 | NRQ | 81.30 | [28] |
|  |  |  | NRK | 18.70 |  |
| Derivata Di Siria | Italy | 157 | NRQ | 85.00 | [29] |
|  |  |  | NRK | 15.00 |  |
| Pantellaria | Italy | 56 | NRQ | 77.00 | [29] |
|  |  |  | NQQ | 23.00 |  |
| Damascus | Cyprus | 219 | NRQ | 87.00 | [31] |
|  |  |  | SRQ | 7.30 |  |
|  |  |  | DRQ | 5.70 |  |
| Damascus | Turkey | 32 | NRQ | 71.90 | [32] |
|  |  |  | SRQ | 28.10 |  |
| Akkeci | Turkey | 16 | NRQ | 87.50 | [32] |
|  |  |  | NQQ | 12.50 |  |
| Saanen | Turkey | 44 | NRQ | 89.80 | [32] |
|  |  |  | DRQ | 2.30 |  |
|  |  |  | NQQ | 4.50 |  |
|  |  |  | NRK | 3.40 |  |
| Kilis | Turkey | 40 | NRQ | 92.50 | [32] |
|  |  |  | DRQ | 7.50 |  |
| Alpine | France | 220 | NRQ | 85.50 | [13] |
|  |  |  | NQQ | 7.10 |  |
|  |  |  | NRK | 7.40 |  |
| Saanen | France | 184 | NRQ | 76.60 | [13] |
|  |  |  | NQQ | 18.50 |  |
|  |  |  | NRK | 4.90 |  |
| Dairy breeds | UK | 932 | NRQ | 96.80 | [30] |
|  |  |  | SRQ | 0.20 |  |
|  |  |  | NQQ | 2.30 |  |
|  |  |  | NRK | 0.70 |  |
| Alpine | USA | 36 | NRQ | 88.90 | [26] |
|  |  |  | SRQ | 1.40 |  |
|  |  |  | NQQ | 9.70 |  |
| Oberhasli | USA | 31 | NRQ | 93.50 | [26] |
|  |  |  | NQQ | 6.50 |  |
| Toggenburg | USA | 37 | NRQ | 91.90 | [26] |
|  |  |  | NQQ | 2.70 |  |
|  |  |  | NRK | 5.40 |  |
| LaMancha | USA | 50 | NRQ | 74.00 | [26] |
|  |  |  | SRQ | 21.00 |  |
|  |  |  | NQQ | 4.00 |  |
|  |  |  | NRK | 1.00 |  |
| Nubian | USA | 47 | NRQ | 67.00 | [26] |
|  |  |  | SRQ | 33.00 |  |
| Saanen | USA | 83 | NRQ | 94.60 | [26] |
|  |  |  | SRQ | 1.80 |  |
|  |  |  | NQQ | 3.60 |  |
| Boer | China | 30 | NRQ | 42.60 | [24] |
|  |  |  | SRQ | 57.40 |  |
| Saanen | Japan | 118 | NRQ | 85.60 | [25] |
|  |  |  | SRQ | 1.70 |  |
|  |  |  | NQQ | 12.70 |  |
