# Supplementary material for: Genetic profile of scrapie codons 146, 211 and 222 in the PRNP gene locus in three breeds of dairy goats
Source: PLoS One. 2018 Jun 7;13(6):e0198819. doi: 10.1371/journal.pone.0198819 (PMC5991713; doi:10.1371/journal.pone.0198819)
Supplement: S2 Table — (DOCX) [file pone.0198819.s003.docx]

**S2 Table. Haplotypic frequencies (%) at the *PRNP* gene locus (codon order 146, 211, 222).**

|  | **Breed** | | | |
| --- | --- | --- | --- | --- |
| **Observed Haplotype** | **Eghoria** | **Skopelos** | **Damascus** | **Total** |
| NRQ | 92.42 | 93.91 | 90.22 | 93.20 |
| SRQ | 0.00 | 0.17 | 6.05 | 1.83 |
| DRQ | 0.19 | 0.00 | 0.00 | 0.06 |
| NQQ | 1.52 | 0.00 | 3.26 | 1.44 |
| NRK | 5.87 | 5.92 | 0.47 | 4.37 |
